# Editorial

**Published:** 1871-08

**Authors:** 


### Advertising Department.

### EDITORIAL.

An advertising department, to meet the wants of the Medical Profession, is a necessity in a Medical Journal—hence we propose in this department, to devote a sufficient amount of space as, from time to time, shall amply serve the purpose of the Profession, by calling their attention to the very best houses in the country from which they may feel perfectly safe in purchasing their drugs, chemicals, etc. Much of the physician's success depends upon the reliability and purity of the medicines he prescribes. His duty is to *know* that his medicines are pure. His own life and that of his loved ones, may depend upon this fact. In order that he may feel confident as to the purity of the medicines administered by him, he must, if he dispenses them himself, purchase *alone* from honorable, reliable parties; if the apothecary fill his bills or prescriptions, he should be requested and instructed to keep on hand for this purpose, the drugs, preparations, etc., manufactured by houses designated by him. In other words, the physician should refuse to patronize any druggist who would not cheerfully put himself to the trouble of complying with such a request. And, above all, the physician should, in making purchases of drugs, not allow inferior articles to be palmed off upon him because of being cheap. Let him bear in mind, and so instruct his patrons, that cheap drugs are an abomination. Loss of life, to say nothing of reputation, results directly from cheap drugs—drugs purporting to be pure, but which are villainous adulterations.

Our publisher has left the advertising columns to the absolute control of the Editors, in order that no party or house shall be allowed a place in them, who are not perfectly reliable. We have refused to allow parties whom we could not endorse, to appear in our advertising pages. We solicit none to advertise but those whom we can endorse and concienciously recommend to the Profession, and to Southern druggists; as our object is to serve the Profession, and in this way confer good, and good only upon our people. By this, too, houses of undoubted integrity, will be brought prominently before the Medical Profession of the South. Hence the honest manufacturer of, or dealer in, drugs, etc.; the honest physician and our honorable publisher will all be mutually interested and benefitted. We, therefore, respectfully ask our readers to examine and become familiar with our advertising columns—by this means they will familiarize themselves with the best drug firms in the country, and learn who of them desire the patronage of Southern physicians.

TILDEN & Co., New Lebanon, N. Y., and 176, William St., New York City. We call the attention of our readers to the old and strictly honorable firm, whose name heads this notice. As manufacturing Pharmaceutists and Chemists, the Messrs. Tilden & Co. take first-rank in this or any other country. With commendable energy they are constantly bringing new remedies, fine chemicals, and preparations before the Profession. In proof of this we refer to their advertisement, in our advertising columns. We have used, with very great advantage, some of their preparations, many of which we propose to speak of in the future.

MESSRS. SHARP & DOHME, Chemists, Baltimore, Md. This excellent house has been represented in our Journal since its first issue. A number of the preparations manufactured by them were left in our hands, which, upon trial, we found reliable and exactly as represented. Messrs. Redwine & Fox, of this city, are their agents.

WM. R. WARNER & Co., Manufacturers of Sugar-Coated Pills, 154, North Third Street, Philadelphia, Pa. This is one of the most extensive and reliable manufacturing houses, North. Their large list of Sugar-Coated Pills will be found, upon trial, strictly reliable and pure, in all instances. We have used the Pil: Iodoform et Ferri, with great advantage. We believe, too, we were first in this city to have the "Live Drug Store," of Messrs. Redwine & Fox, keep on hand a supply of the Pil: Phosphorus Comp., which we have prescribed, with very decided advantage, in appropriate cases. Messrs. Redwine & Fox, of this city, are the agents.

